# Selective Autophagy: A Link Across the Hallmarks of Aging

**DOI:** 10.1093/geroni/igab046.1971

**Published:** 2021-12-17

**Authors:** Ana Maria Cuervo

**Affiliations:** Albert Einstein College of Medicine, Bronx, New York, United States

## Abstract

Autophagy function has been closely linked with the loss of proteostasis that characterizes most old organisms and tissues. However, the cellular functions of selective types of autophagy such as chaperone-mediated autophagy (CMA) go beyond cellular quality control. CMA can degrade fully functional proteins to terminate their function and thus contribute to regulation of multiple cellular processes. To fully understand the consequences of loss of CMA function with age, we have developed genetic and pharmacological ways to modulate this pathway in old mice. Our data supports involvement of CMA in other hallmarks of aging such as metabolism, senescence, cellular response to stress, epigenetics and cellular stemness. This interconnection among the cellular processes that drive aging highlights the potential of acting on only some of them with geroprotective effects.

